# A comparative study of fruit and vegetable consumption and physical activity among adolescents in 49 Low-and-Middle-Income Countries

**DOI:** 10.1038/s41598-018-19956-0

**Published:** 2018-01-26

**Authors:** Sandra A. Darfour-Oduro, David M. Buchner, Juan E. Andrade, Diana S. Grigsby-Toussaint

**Affiliations:** 1 Department of Kinesiology and Community Health, University of Illinois at Urbana-Champaign, Champaign, USA; 2 Division of Nutritional Sciences, University of Illinois at Urbana-Champaign, Urbana, USA; 3 Department of Food Science and Human Nutrition, University of Illinois at Urbana-Champaign, Urbana, USA

## Abstract

Physical inactivity and low consumption of fruits and vegetables (FV) during adolescence may persist through adulthood, putting adolescents at risk of developing chronic diseases. Although studies from high-income countries have reported differences in FV consumption and physical activity (PA) between adolescent boys and girls, few exist from low-and-middle-income countries (LMICs). In this study, we examined patterns of FV consumption and PA among adolescent boys and girls in LMICs. Country selection was based on availability of Global School-Based Student Health Survey (GSHS) data from 2004 to 2013. The total analytic sample was 164,771 adolescents from 49 LMICs. Descriptive statistics were generated to determine adolescents meeting the World Health Organization (WHO) recommendations for FV and PA. A Rao-Scott adjusted chi-square statistic was computed to assess gender differences. Less than 30% of adolescents across all countries met the WHO guidelines for FV consumption or PA. Morocco (29.5%) and India (29.5%) however had the highest percentage of adolescents meeting recommendations for FV and PA, respectively. Adolescent boys were more active than girls, and this difference was more notable in the Middle East and North African region. Adolescents achieving the WHO recommendations for daily consumption of FV and PA were consistently low in all countries.

## Introduction

Non-communicable diseases (NCDs), such as cancer, diabetes, and cardiovascular disease, are the leading cause of death worldwide^1^. Currently, NCDs are responsible for about 70% of deaths^1^. NCDs are especially important in low- and-middle-income countries, where more than three-quarters of NCD deaths occur^1^. It is well known that risk of NCDs is primarily related to four behavioral risk factors: tobacco use, physical inactivity, an unhealthy diet, and excessive alcohol use^2,3^. It is a public health priority to promote healthy lifestyles as well as monitor levels of behavioral risk factors in all age groups, including children and adolescents. For this purpose, the WHO has established recommendations for adolescents for minimal levels of physical activity (PA) and dietary intake of fruits and vegetables (FV). Currently, the WHO recommends at least 60 minutes of moderate-to-vigorous physical activity each day^4^, and a dietary intake of at least 400 grams of FV each day (roughly equivalent to five servings of FV/day)^5^.

Despite these WHO recommendations, current evidence indicates that adolescents eat far less than the recommended five daily servings of FV. For example, a study by Peltzer & Pengpid^6^ in seven African countries (Botswana, Kenya, Senegal, Swaziland, Tanzania, Uganda, and Zambia) found that most adolescents (77.5%) did not meet the recommended daily servings of FV. Similarly, Doku *et al*.^7^ found that 56% and 48% of adolescents in Ghana rarely (≤3 days/week) consumed fruits and vegetables respectively. In China, Shi *et al*.^8^ found that less than 50% of school children ate fruits daily. Also, in the North Gaza strip of Palestinian Territories, Abudayya *et al*.^9^ found that only 11.6% of boys and 16.2% of girls consumed fruits daily. This shows that FV consumption is consistently low in many LMICs.

Studies have also shown gender differences concerning the daily intake of FV. For example, in a review of determinants of FV intake among children and adolescents, Rasmussen *et al*.^10^ found that of the 49 studies reviewed, 27 studies found that girls had a higher consumption of fruits and vegetables compared to boys. In 18 studies, there were no differences in the intake of FV between adolescents’ boys and girls. Lastly, in four studies, the authors found that boys had a higher FV intake compared to girls^10^.

Also, it appears children and youth between the ages of 5–17 commonly do not engage in at least 60 minutes of moderate- to vigorous-intensity PA daily^4^. Moreover, studies have shown that PA declines during adolescence, especially among girls^11,12^. In an examination of PA patterns among adolescents using the Global School-based Student Health Survey (GSHS) and the Health Behavior in School-aged Children (HBSC) survey, Hallal *et al*.^13^ found that 80.3% of adolescents between 13–15 years from 105 countries did not meet the recommended daily PA. The authors also found that girls were less active than boys^13^.

Similarly, in 2010, the WHO found that globally 81% of adolescents aged 11−17 did not meet the daily physical activity recommendation^14^. The study results showed that adolescent girls recorded a higher percentage (84%) of insufficient PA compared to adolescent boys (78%)^14^. When disaggregating the PA data by WHO regions, the Eastern Mediterranean Region (88%), the African Region (85%) and the Western Pacific Region (85%) were ranked highest among regions reporting insufficient PA among adolescents. In all WHO regions, adolescent girls were less active than boys^14^. Thus, the adolescence represents a critical period to encourage and create a conducive environment to be active, thereby minimizing the risk of NCDs, especially among girls.

However, there are few cross-national studies from LMICs examining patterns of FV consumption and physical activity among adolescent boys and girls. The purpose of this study was to compare FV consumption and PA behavior patterns among adolescent girls and boys in 49 LMICs. We hypothesize that there are differences between the FV consumption and PA behavior patterns among adolescent girls and boys in LMICs.

## Methods

### Study design and sampling method

This study utilizes an ecologic study design to compare patterns of FV consumption and PA among adolescents from countries and regions demarcated by the World Bank as LMICs. The World Bank regions are Sub-Saharan Africa, Middle East and North Africa, South Asia, Latin America and the Caribbean, Europe and Central Asia and East Asia and the Pacific^15^. The study sample of LMICs included countries from the regions demarcated by the World Bank if they had (1) publicly available data on FV intake and PA behaviors), and (2) data available to allow the assessment of differences between adolescent boys and girls for these health behaviors.

### Data and Sample

The primary data source is the Global School-based Student Health Survey (GSHS). The GSHS is a standardized survey developed by the WHO in collaboration with the United Nations International Children’s Emergency Fund, the United Nations Educational, Scientific and Cultural Organization, and the United Nations Program on HIV/AIDS, with technical assistance provided by the Centers for Disease Control and Prevention. The GSHS is a collaborative surveillance project designed to help countries measure and assess health behaviors and protective factors among students between the ages of 13 to 17 years. It allows countries to develop priorities, establish programs, and advocate for resources for youth health programs and policies^16^.

**Figure 1:**
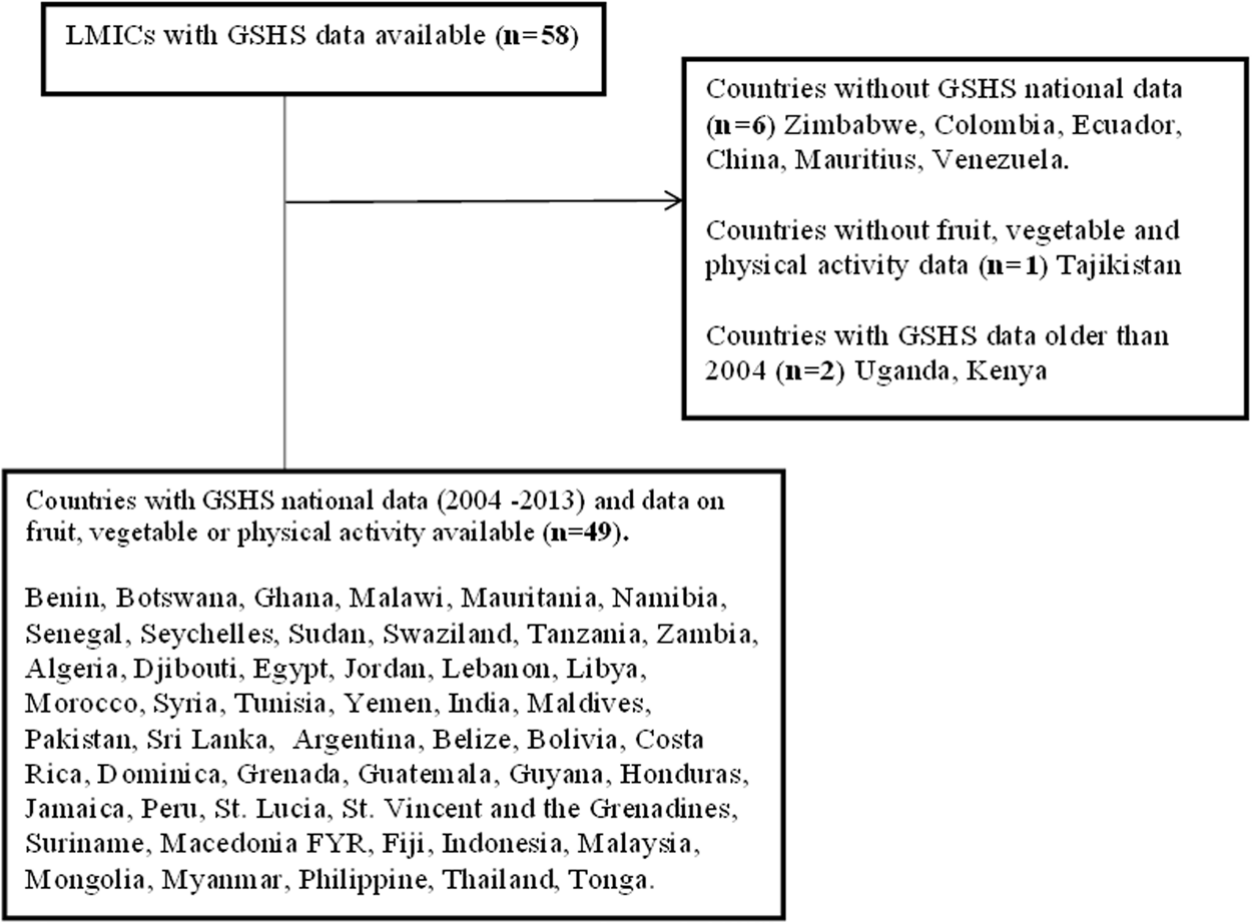
Selection process for LMICs based on available GSHS national data between 2004–2013.

As of December 2013, 90 countries (mostly LMICs) had completed the GSHS, and representatives from over 120 countries have been trained to administer the GSHS^16^. GSHS data from 2004 to 2013 that were publicly available for LMICs^15^ were used. According to the World Bank, these are countries that had a gross national income (GNI) of less than $12,616 in 2012^15^. A total of 49 countries with publicly available data on dietary behaviors and PA for adolescents met this definition for inclusion in our study. Figure 1 shows the LMICs included in the study (n = 49 countries).

### Survey Measures

The survey measures used were based on the following GSHS questions^17^:

**Fruit**; i) “During the past 30 days, how many times per day did you usually eat fruits, such as COUNTRY SPECIFIC EXAMPLES?” Response options were; (A) I did not eat fruit during the past 30 days (B) Less than one time per day (C) 1 time per day (D) 2 times per day (E) 3 times per day (F) 4 times per day (G) 5 or more times per day.

**Vegetables**; ii) “During the past 30 days, how many times per day did you usually eat vegetables, such as COUNTRY SPECIFIC EXAMPLES?”The response options were (A) I did not eat vegetables during the past 30 days (B) Less than one time per day (C)1 time per day (D) 2 times per day (E) 3 times per day (F) 4 times per day (G)5 or more times per day.

**Physical Activity**; iii) “During the past 7 days, on how many days were you physically active for a total of at least 60 minutes per day?” The response options; A) 0 days B) 1 day C) 2 days D) 3 days E) 4 days F) 5 days G) 6 days H) 7 days.

### Data analysis

The data were analyzed using SPSS version 21 software (SPSS, Inc., Chicago, IL). The GSHS employs a complex sampling design, and the data were analyzed taking into account this sampling design. The weighting process was also used. This process adds weights, stratum and primary sampling unit (PSU) to every student record in the GSHS data file to reflect the weighting process and the 2-staged sampling design. The weight variable allows generalizability of the GSHS results to the entire students’ population whereas the stratum and PSU account for the 2-stage sample design used for the GSHS. Specifically, the stratum reflects the GSHS sampling process at the first level, which is done in schools and the PSU reflects the second level of the GSHS sampling process conducted in classrooms^18^.

Descriptive statistics were generated to determine the number and percentage of adolescents in each of the LMICs meeting the WHO recommendations of daily intake of FV and PA. In generating the descriptive statistics, the variables took values of 1 (two otherwise) for i) consumption of fruits: adequate (2 or more/day), ii) consumption of vegetables: adequate (3 or more/day), iii) consumption of FV: adequate (adequate on both FV), iv) PA: adequate (60 minutes/day).The Rao-Scott adjusted chi-square statistic was used to test if there was a statistically significant difference between adolescent boys and girls in meeting the WHO recommendations of daily fruit servings, daily vegetable servings, daily FV servings and daily PA for each country and odds ratios were also calculated. The Rao-Scott adjusted chi-square statistic takes into consideration the complex sample design utilized in the GSHS data collection. The p-value of the adjusted F- statistics generated was used in determining statistical significance because it provides a better approximation of the significance^19^.

### Availability of data and material

The datasets generated and/or analyzed during the current study are available at http://www.who.int/chp/gshs/datasets/en/.

## Results

**Figure 2:**
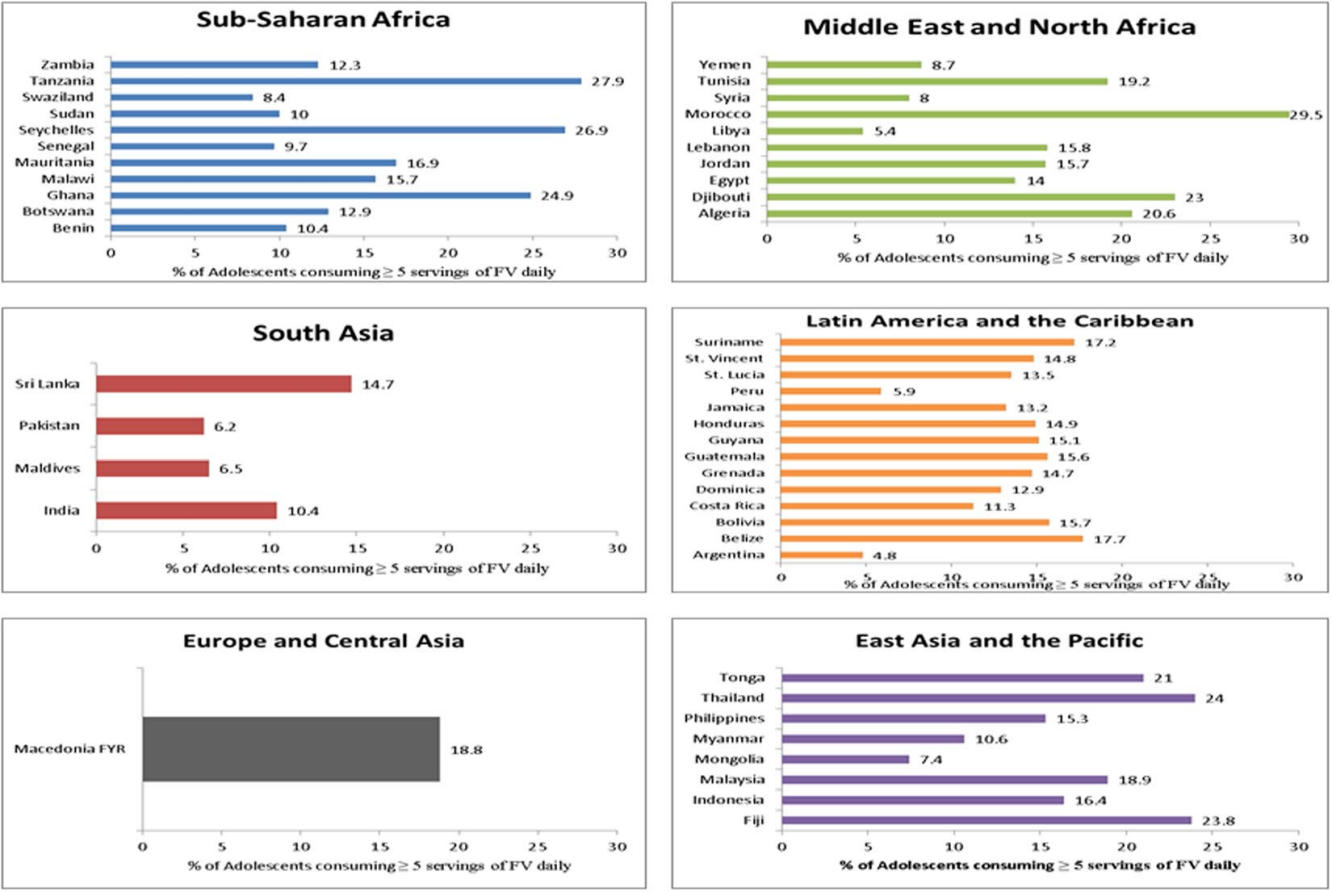
Percentage of adolescents meeting daily FV intake (5 servings) in LMICs grouped by World Bank regions.

**Figure 3:**
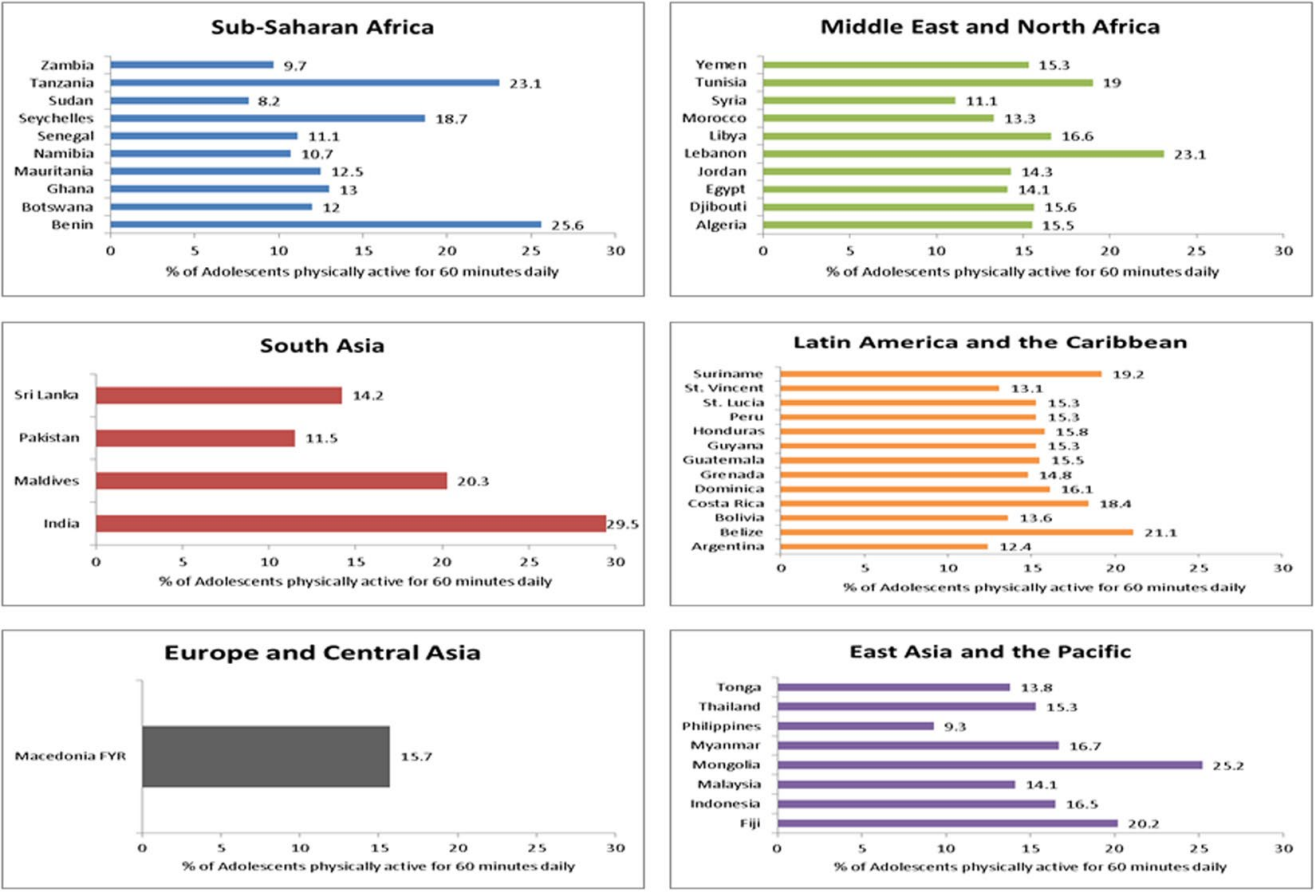
Percentage of adolescents meeting the daily PA recommendations in LMICs grouped by World Bank regions.

Figure 2 summarizes the pattern of FV consumption and PA among adolescents in LMICs grouped by World Bank regions. The sample represented 164,771 adolescents from 49 LMICs. There was significant variability regarding the outcomes of interest throughout the different regions and countries included in this study. In all countries, less than 50% of adolescents consumed five or more FV per day (Fig. 2). Morocco had the highest proportion of adolescents consuming the recommended daily FV intake (29.5%). Lastly, adolescents in India were the most active (29.5%) in terms of meeting the WHO recommendation for daily PA (Fig. 3).

Differences in the outcomes of interest between adolescent boys and girls were observed in some but not all countries. In the sub-Saharan African region, only two countries (Senegal and Swaziland) showed girls more likely to meet the daily recommended intake of fruits compared to boys. Similarly, in two other countries (Seychelles and Tanzania) girls were more likely (p < 0.05) to meet the daily recommended intake of vegetables (Table 1

**Table 1:** Differences in the daily recommended intake of fruits and vegetables among adolescent girls and boys in Low-and Middle-Income Countries from the Global School-based Student Health Survey (2004–2013) (N = 163,123 adolescents from 49 LMICs).

**LMICs**	Fruit servings recommendations					Vegetable servings recommendations			
		≥per 2 day	<2 per day	Adjusted F (p-value)	OR (95% CI)	≥3 per day	<3 per day	Adjusted F (p-value)	OR (95% CI)
		N (weighted %)				N (weighted %)			
**Sub-Saharan Africa**									
Benin	Girls	309 (35.5)	622 (64.5)	0.351	1.16 (0.84–1.61)	171 (18.9)	757 (81.1)	0.338	1.18 (0.83–1.68)
	Boys	520 (32.1)	1219 (67.9)			274 (16.5)	1465 (83.5)		
Botswana	Girls	473 (39.7)	717 (60.3)	0.267	1.09 (0.93–1.28)	278 (23.4)	905 (76.6)	0.139	1.19 (0.94–1.52)
	Boys	367 (37.7)	606 (62.3)			197 (20.4)	762 (79.6)		
Ghana	Girls	1417 (50.6)	1467 (49.4)	0.352	1.08 (0.92–1.27)	1029 (37.8)	1865 (62.2)	0.557	1.04 (0.90–1.21)
	Boys	1499 (48.7)	1678 (51.3)			1111 (36.8)	2070 (63.2)		
Malawi	Girls	668 (52.1)	529 (47.9)	0.865	0.98 (0.78–1.24)	327 (24.3)	882 (75.7)	0.710	1.07 (0.75–1.53)
	Boys	557 (52.5)	490 (47.5)			262 (23.1)	786 (76.9)		
Mauritania	Girls	360 (34.0)	693 (66.0)	0.237	1.20 (0.87–1.64)	272 (26.3)	764 (73.7)	0.503	0.90 (0.63–1.27)
	Boys	280 (30.1)	669 (69.9)			261 (28.5)	675 (71.5)		
Namibia	Girls	—	—	—	—	—	—	—	—
	Boys	—	—			—	—		
Senegal	Girls	485 (35.9)	910 (64.1)	**0.008**	**1.31 (1.09–1.58)**	211 (15.2)	1187 (84.8)	0.290	0.91 (0.75–1.10)
	Boys	505 (30.0)	1190 (70.0)			280 (16.5)	1419 (83.5)		
Seychelles	Girls	435 (58.8)	301 (41.2)	0.269	0.97 (0.92–1.02)	262 (35.3)	474 (64.7)	**0.012**	**1.07 (1.02–1.13)**
	Boys	404 (59.5)	272 (40.5)			228 (33.8)	446 (66.2)		
Sudan	Girls	357 (27.4)	960 (72.6)	0.238	1.23 (0.86–1.76)	240 (18.2)	1088 (81.8)	0.539	0.89 (0.60–1.31)
	Boys	198 (23.4)	639 (76.6)			161 (20.0)	675 (80.0)		
Swaziland	Girls	730 (38.7)	1167 (61.3)	**0.032**	**1.27 (1.02–1.56)**	278 (15.1)	1620 (84.9)	0.455	1.09 (0.86–1.36)
	Boys	565 (33.3)	1153 (66.7)			234 (14.1)	1495 (85.9)		
Tanzania	Girls	597 (53.3)	508 (46.7)	0.144	1.13 (0.95–1.35)	446 (41.3)	657 (58.7)	**0.036**	**1.32 (1.02–1.70)**
	Boys	503 (50.2)	518 (49.8)			354 (34.8)	668 (65.2)		
Zambia	Girls	469 (44.5)	598 (55.5)	0.639	1.06 (0.83–1.34)	213 (20.6)	852 (79.4)	0.309	0.89 (0.69–1.13)
	Boys	428 (43.2)	588 (56.8)			208 (22.7)	807 (77.3)		
**Middle East and North Africa**									
Algeria	Girls	1258 (57.5)	1055 (42.5)	**0.000**	**1.49 (1.30–1.71)**	715 (30.5)	1601 (69.5)	0.758	1.03 (0.86–1.24)
	Boys	950 (47.5)	1190 (52.5)			637 (29.9)	1504 (70.1)		
Djibouti	Girls	362 (47.5)	398 (52.5)	**0.020**	**1.26 (1.04–1.52)**	273 (36.1)	484 (63.9)	0.357	1.12 (0.87–1.44)
	Boys	424 (41.8)	579 (58.2)			338 (33.6)	663 (66.4)		
Egypt	Girls	726 (54.0)	606 (46.0)	0.918	1.03 (0.61–1.74)	261 (18.8)	1076 (81.2)	0.782	1.10 (0.54–2.24)
	Boys	644 (53.3)	510 (46.7)			220 (17.4)	941 (82.6)		
Jordan	Girls	372 (39.1)	578 (60.9)	0.996	1.00 (0.67–1.49)	224 (24.0)	728 (76.0)	0.581	0.91 (0.62–1.33)
	Boys	477 (39.0)	731 (61.0)			320 (25.9)	897 (74.1)		
Lebanon	Girls	588 (47.2)	629 (52.8)	**0.002**	**0.74 (0.63–0.88)**	231 (18.8)	986 (81.2)	**0.034**	**0.76 (0.59–0.98)**
	Boys	600 (54.6)	463 (45.4)			241 (23.4)	820 (76.6)		
Libya	Girls	255 (20.5)	987 (79.5)	0.824	1.02 (0.83–1.27)	151 (12.1)	1102 (87.9)	0.42	0.89 (0.66–1.19)
	Boys	190 (20.2)	751 (79.8)			123 (13.4)	820 (86.6)		
Morocco	Girls	813 (59.5)	551 (40.5)	**0.000**	**1.61 (1.39–1.85)**	590 (44.1)	755 (55.9)	**0.010**	**1.28 (1.07–1.53)**
	Boys	722 (47.7)	784 (52.3)			567 (38.1)	925 (61.9)		
Syria	Girls	639 (35.5)	1207 (64.5)	0.245	1.13 (0.92–1.40)	214 (11.5)	1639 (88.5)	0.285	0.86 (0.65–1.14)
	Boys	405 (32.8)	824 (67.2)			165 (13.2)	1076 (86.8)		
Tunisia	Girls	815 (56.5)	626 (43.5)	**0.008**	**1.34 (1.10–1.65)**	350 (24.4)	1092 (75.6)	**0.000**	**0.66 (0.56–0.77)**
	Boys	672 (49.2)	697 (50.8)			447 (33.0)	917 (67.0)		
Yemen	Girls	67 (16.4)	349 (83.6)	0.295	0.72 (0.37–1.39)	57 (13.8)	361 (86.2)	0.463	0.77 (0.37–1.62)
	Boys	141 (21.6)	500 (78.4)			113 (17.2)	538 (82.8)		
**South Asia**									
India	Girls	953 (28.1)	2584 (71.9)	0.613	1.05 (0.88–1.25)	938 (24.9)	2605 (75.1)	0.769	0.97 (0.79–1.19)
	Boys	1154 (27.2)	3308 (72.8)			1166 (25.4)	3282 (74.6)		
Maldives	Girls	318 (19.3)	1420 (80.7)	**0.002**	**0.67 (0.52–0.86)**	123 (7.7)	1612 (92.3)	**0.001**	**0.58 (0.42–0.80)**
	Boys	354 (26.4)	1085 (73.6)			164 (12.6)	1263 (87.4)		
Pakistan	Girls	335 (25.5)	948 (74.5)	0.124	1.52 (0.88–2.60)	163 (12.9)	1122 (87.1)	**0.018**	**0.48 (0.27–0.88)**
	Boys	702 (18.4)	3178 (81.6)			889 (23.4)	2994 (76.6)		
Sri Lanka	Girls	491 (34.2)	960 (65.8)	0.285	1.17 (0.87–1.58)	468 (32.0)	987 (68.0)	0.459	1.11 (0.82–1.51)
	Boys	336 (30.7)	791 (69.3)			324 (29.7)	802 (70.3)		
**Latin America and the Caribbean**									
Argentina	Girls	342 (34.6)	651 (65.4)	0.053	1.27 (1.00–1.63)	87 (9.2)	897 (90.8)	0.199	1.22 (0.90–1.65)
	Boys	285 (29.4)	669 (70.6)			77 (7.7)	873 (92.3)		
Belize	Girls	513 (46.4)	595 (53.6)	0.276	0.88 (0.70–1.12)	282 (25.5)	826 (74.5)	0.605	1.07 (0.83–1.38)
	Boys	478 (49.5)	510 (50.5)			237 (24.3)	750 (75.7)		
Bolivia	Girls	804 (46.2)	924 (53.8)	0.778	1.02 (0.90–1.15)	406 (23.2)	1317 (76.8)	0.177	0.87 (0.71–1.07)
	Boys	819 (45.8)	963 (54.2)			467 (25.8)	1317 (74.2)		
Costa Rica	Girls	401 (29.2)	968 (70.8)	**0.039**	**0.80 (0.65–0.99)**	235 (17.4)	1143 (82.6)	0.114	0.85 (0.69–1.05)
	Boys	438 (34.1)	845 (65.9)			255 (19.9)	1030 (80.1)		
Dominica	Girls	383 (41.8)	538 (58.2)	**0.014**	**0.74 (0.58–0.94)**	149 (16.4)	769 (83.6)	0.155	0.83 (0.64–1.08)
	Boys	343 (49.3)	361 (50.7)			135 (19.2)	572 (80.8)		
Grenada	Girls	433 (52.5)	403 (47.5)	0.085	0.80 (0.62–1.03)	169 (20.6)	667 (79.4)	0.621	1.09 (0.77–1.53)
	Boys	393 (58.1)	286 (41.9)			139 (19.2)	539 (80.8)		
Guatemala	Girls	1284 (42.2)	1703 (57.8)	0.977	1.00 (0.70–1.42)	640 (21.1)	2339 (78.9)	0.725	0.97 (0.81–1.16)
	Boys	1038 (42.4)	1463 (57.6)			529 (21.7)	1938 (78.3)		
Guyana	Girls	601 (45.2)	714 (54.8)	0.050	0.77 (0.59–1.00)	300 (23.7)	1024 (76.3)	0.996	1.00 (0.75–1.34)
	Boys	524 (51.7)	489 (48.3)			243 (23.7)	784 (76.3)		
Honduras	Girls	382 (42.5)	525 (57.5)	0.857	0.98 (0.78–1.23)	177 (19.7)	722 (80.3)	0.430	0.91 (0.70–1.17)
	Boys	356 (42.9)	478 (57.1)			168 (21.3)	659 (78.7)		
Jamaica	Girls	327 (40.4)	483 (59.6)	**0.007**	**0.63 (0.47–0.86)**	127 (18.9)	675 (81.1)	0.567	0.88 (0.55–1.41)
	Boys	400 (51.7)	373 (48.3)			142 (20.9)	621 (79.1)		
Peru	Girls	509 (34.2)	953 (65.8)	**0.014**	**1.26 (1.05–1.51)**	116 (7.9)	1345 (92.1)	0.218	0.80 (0.55–1.15)
	Boys	415 (29.2)	986 (70.8)			137 (9.8)	1261 (90.2)		
St. Lucia	Girls	299 (41.1)	428 (58.9)	0.566	0.94 (0.74–1.18)	143 (20.5)	576 (79.5)	0.285	1.14 (0.90–1.44)
	Boys	234 (42.8)	300 (57.2)			104 (18.5)	421 (81.5)		
St. Vincent and the Grenadines	Girls	398 (58.3)	294 (41.7)	0.493	0.93 (0.74–1.16)	110 (17.3)	575 (82.7)	0.348	0.83 (0.55–1.24)
	Boys	370 (60.2)	251 (39.8)			123 (20.2)	493 (79.8)		
Suriname	Girls	356 (43.1)	469 (56.9)	0.105	0.86 (0.71–1.04)	227 (27.7)	598 (72.3)	0.926	0.99 (0.78–1.26)
	Boys	408 (46.9)	454 (53.1)			241 (27.9)	622 (72.1)		
**Europe and Central Asia**									
The Former Yugoslav Republic of Macedonia	Girls	613 (59.7)	437 (40.3)	0.682	0.95 (0.75–1.22)	220 (22.1)	826 (77.9)	0.809	1.04 (0.74–1.46)
	Boys	605 (60.9)	412 (39.1)			219 (21.4)	809 (78.6)		
**East Asia and Pacific**									
Fiji	Girls	427 (47.6)	518 (52.4)	0.693	0.95 (0.72–1.25)	330 (36.3)	621 (63.7)	0.646	1.08 (0.76–1.54)
	Boys	319 (48.9)	386 (51.1)			248 (34.5)	462 (65.5)		
Indonesia	Girls	622 (39.5)	995 (60.5)	0.268	1.09 (0.93–1.29)	491 (30.7)	1118 (69.3)	0.326	1.10 (0.91–1.32)
	Boys	545 (37.4)	928 (62.6)			435 (28.8)	1032 (71.2)		
Malaysia	Girls	5617 (44.1)	7086 (55.9)	0.873	1.01 (0.93–1.09)	3531 (28.0)	9185 (72.0)	**0.000**	**0.85 (0.78–0.91)**
	Boys	5621 (43.9)	7071 (56.1)			3919 (31.5)	8797 (68.5)		
Mongolia	Girls	464 (16.2)	2376 (83.8)	0.079	0.88 (0.76–1.02)	639 (22.2)	2192 (77.8)	**0.019**	**0.83 (0.71–0.97)**
	Boys	465 (18.1)	2038 (81.9)			656 (25.7)	1840 (74.3)		
Myanmar	Girls	374 (26.9)	1030 (73.1)	0.284	1.13 (0.90–1.42)	306 (21.7)	1095 (78.3)	0.644	1.07 (0.80–1.44)
	Boys	340 (24.6)	1050 (75.4)			272 (20.6)	1122 (79.4)		
Philippines	Girls	1097 (38.8)	1859 (61.2)	0.546	1.04 (0.91–1.20)	709 (25.3)	2266 (74.7)	0.548	0.94 (0.75–1.17)
	Boys	804 (37.8)	1414 (62.2)			563 (26.5)	1706 (73.5)		
Thailand	Girls	682 (48.3)	708 (51.7)	0.207	1.13 (0.93–1.37)	496 (35.5)	899 (64.5)	0.208	0.87 (0.69–1.09)
	Boys	603 (45.3)	752 (54.7)			523 (38.9)	841 (61.1)		
Tonga	Girls	507 (43.2)	667 (56.8)	0.248	1.15 (0.91–1.45)	458 (38.5)	716 (61.5)	0.144	1.21 (0.94–1.57)
	Boys	405 (39.9)	600 (60.1)			332 (34.1)	650 (65.9)		

OR = Odds Ratio; CI = Confidence Interval; Boldface value are significant at P < 0.05; - no data available.

).

In the Middle East and North African region, a significant difference was observed in the daily intake of fruits among adolescent boys and girls in 50% of the countries examined. In four (Algeria, Djibouti, Morocco and Tunisia) out of the five countries, adolescent girls were more likely to meet the daily recommended servings of fruit as compared to boys. In Lebanon, however, adolescent girls were less likely to consume two servings of fruits daily compared to adolescent boys {OR = 0.74; 95%CI (0.63–0.88); p-value = 0.002} (Table 1).

With regards to daily servings of vegetables (≥3 per day), a statistically significant difference between adolescent girls and boys was seen in three countries in the Middle East and North Africa. In two of the three countries (Lebanon and Tunisia), adolescent girls were less likely to consume ≥3 servings of vegetables per day than boys. Nonetheless, in Morocco {OR = 1.28; 95%CI (1.07–1.53); p-value = 0.01}, girls were more likely to consume the daily recommended intake of vegetables compared to boys (Table 1). In six countries there were significant differences between adolescent girls and boys meeting the recommended five servings of FV daily. In four countries (Lebanon, Syria, Tunisia, and Yemen), adolescent girls were less likely to consume the daily recommended servings of FV compared to boys. However, in Algeria {OR = 1.23; 95%CI (1.03–1.48); p-value = 0.026} and Morocco {OR = 1.31; 95%CI (1.08–1.60); p-value = 0.01}, girls were more likely to have five or more servings of FV daily than boys (Table 2).

**Table 2:** Differences in the recommended daily fruit and vegetable intake among adolescent girls and boys in Low-and Middle-Income Countries from the Global School-based Student Health Survey (2004–2013) (N = 163,123 adolescents from 49 LMICs).

**LMICs**		Fruit & vegetable (FV) servings recommendations			
		>=5 servings per day	<5 servings per day	Adjusted F (p-value)	OR (95% CI)
		N (weighted %)			
**Sub-Saharan Africa**					
Benin	Girls	102 (11.4)	826 (88.6)	0.488	1.16 (0.75–1.80)
	Boys	159 (10.0)	1579 (90.0)		
Botswana	Girls	164 (13.9)	1014 (86.1)	0.082	1.19 (0.98–1.44)
	Boys	116 (11.9)	841 (88.1)		
Ghana	Girls	690 (25.7)	2178 (74.3)	0.376	1.09 (0.90–1.30)
	Boys	737 (24.2)	2423 (75.8)		
Malawi	Girls	216 (15.7)	980 (84.3)	0.997	1.00 (0.66–1.53)
	Boys	174 (15.7)	869 (84.3)		
Mauritania	Girls	168 (16.2)	860 (83.8)	0.637	0.92 (0.64–1.33)
	Boys	156 (17.4)	773 (82.6)		
Namibia	Girls	—	—	—	—
	Boys	—	—		
Senegal	Girls	142 (10.6)	1248 (89.4)	0.209	1.17 (0.91–1.51)
	Boys	153 (9.2)	1536 (90.8)		
Seychelles	Girls	199 (26.9)	534 (73.1)	0.498	1.02 (0.97–1.08)
	Boys	181 (26.6)	493 (73.4)		
Sudan	Girls	128 (9.5)	1179 (90.5)	0.529	0.90 (0.65–1.26)
	Boys	88 (10.4)	742 (89.6)		
Swaziland	Girls	159 (8.7)	1729 (91.3)	0.615	1.08 (0.79–1.47)
	Boys	133 (8.1)	1578 (91.9)		
Tanzania	Girls	324 (30.1)	771 (69.9)	0.087	1.27 (0.96–1.66)
	Boys	249 (25.4)	768 (74.6)		
Zambia	Girls	116 (12.0)	922 (88.0)	0.854	0.97 (0.66–1.41)
	Boys	112 (12.4)	875 (87.6)		
**Middle East and North Africa**					
Algeria	Girls	494 (22.3)	1811 (77.7)	**0.026**	**1.23 (1.03–1.48)**
	Boys	395 (18.8)	1737 (81.2)		
Djibouti	Girls	184 (24.3)	572 (75.7)	0.367	1.13 (0.86–1.49)
	Boys	220 (22.1)	775 (77.9)		
Egypt	Girls	189 (14.3)	1133 (85.7)	0.937	1.03 (0.49–2.15)
	Boys	176 (13.9)	966 (86.1)		
Jordan	Girls	137 (14.5)	812 (85.5)	0.270	0.83 (0.59–1.17)
	Boys	206 (16.9)	998 (83.1)		
Lebanon	Girls	163 (13.0)	1051 (87.0)	**0.001**	**0.64 (0.50–0.83)**
	Boys	196 (18.9)	864 (81.1)		
Libya	Girls	62 (5.0)	1174 (95.0)	0.576	0.89 (0.59–1.35)
	Boys	52 (5.6)	884 (94.4)		
Morocco	Girls	436 (32.6)	905 (67.4)	**0.010**	**1.31 (1.08–1.60)**
	Boys	400 (26.9)	1087 (73.1)		
Syria	Girls	124 (6.8)	1717 (93.2)	**0.037**	**0.73 (0.54–0.98)**
	Boys	113 (9.1)	1114 (90.9)		
Tunisia	Girls	245 (17.3)	1186 (82.7)	**0.017**	**0.78 (0.64–0.95)**
	Boys	287 (21.3)	1071 (78.7)		
Yemen	Girls	23 (5.7)	387 (94.3)	**0.040**	**0.53 (0.29–0.97)**
	Boys	68 (10.2)	566 (89.8)		
**South Asia**					
India	Girls	384 (10.8)	3133 (89.2)	0.605	1.07 (0.82–1.39)
	Boys	454 (10.2)	3980 (89.8)		
Maldives	Girls	78 (4.8)	1649 (95.2)	**0.019**	**0.57 (0.35–0.91)**
	Boys	106 (8.2)	1315 (91.8)		
Pakistan	Girls	68 (5.4)	1213 (94.6)	0.522	0.79 (0.37–1.69)
	Boys	248 (6.7)	3620 (93.3)		
Sri Lanka	Girls	240 (16.7)	1208 (83.3)	0.056	1.38 (0.99–1.93)
	Boys	133 (12.7)	990 (87.3)		
**Latin America and the Caribbean**					
Argentina	Girls	53 (5.5)	931 (94.5)	**0.037**	**1.43 (1.02–2.00)**
	Boys	43 (3.9)	904 (96.1)		
Belize	Girls	195 (17.7)	911 (82.3)	0.957	1.01 (0.75–1.36)
	Boys	170 (17.5)	814 (82.5)		
Bolivia	Girls	269 (15.0)	1445 (85.0)	0.157	0.86 (0.70–1.06)
	Boys	299 (17.0)	1479 (83.0)		
Costa Rica	Girls	134 (10.0)	1234 (90.0)	0.071	0.77 (0.57–1.03)
	Boys	165 (12.6)	1117 (87.4)		
Dominica	Girls	103 (11.8)	812 (88.2)	0.163	0.82 (0.61–1.09)
	Boys	96 (14.0)	604 (86.0)		
Grenada	Girls	122 (14.7)	707 (85.3)	0.959	1.01 (0.69–1.47)
	Boys	103 (14.6)	570 (85.4)		
Guatemala	Girls	458 (15.5)	2511 (84.5)	0.820	0.97 (0.73–1.29)
	Boys	382 (15.9)	2078 (84.1)		
Guyana	Girls	183 (13.9)	1129 (86.1)	0.292	0.86 (0.64–1.16)
	Boys	160 (15.9)	848 (84.1)		
Honduras	Girls	129 (14.6)	768 (85.4)	0.569	0.93 (0.71–1.22)
	Boys	123 (15.5)	701 (84.5)		
Jamaica	Girls	84 (11.5)	712 (88.5)	0.135	0.73 (0.47–1.12)
	Boys	101 (15.1)	659 (84.9)		
Peru	Girls	87 (6.0)	1374 (94.0)	0.710	1.07 (0.74–1.55)
	Boys	80 (5.7)	1318 (94.3)		
St. Lucia	Girls	97 (13.9)	620 (86.1)	0.592	1.08 (0.81–1.45)
	Boys	72 (13.0)	451 (87.0)		
St. Vincent and the Grenadines	Girls	85 (13.5)	598 (86.5)	0.363	0.80 (0.49–1.31)
	Boys	97 (16.3)	515 (83.7)		
Suriname	Girls	125 (15.2)	698 (84.8)	0.053	0.76 (0.57–1.01)
	Boys	166 (19.2)	695 (80.8)		
**Europe and Central Asia**					
The Former Yugoslav Republic of Macedonia	Girls	189 (18.9)	853 (81.1)	0.892	1.02 (0.73–1.43)
	Boys	187 (18.6)	826 (81.4)		
**East Asia and Pacific**					
Fiji	Girls	213 (24.6)	732 (75.4)	0.649	1.10 (0.71–1.71)
	Boys	148 (22.9)	553 (77.1)		
Indonesia	Girls	264 (17.0)	1342 (83.0)	0.553	1.09 (0.81–1.46)
	Boys	233 (15.8)	1231 (84.2)		
Malaysia	Girls	2307 (17.9)	10385 (82.1)	**0.007**	**0.88 (0.79–0.96)**
	Boys	2483 (19.9)	10196 (80.1)		
Mongolia	Girls	205 (7.1)	2615 (92.9)	0.464	0.92 (0.72–1.17)
	Boys	200 (7.7)	2285 (92.3)		
Myanmar	Girls	154 (11.0))	1244 (89.0	0.691	1.09 (0.71–1.68)
	Boys	1262 (89.8)	128 (10.2)		
Philippines	Girls	411 (15.1)	2535 (84.9)	0.793	0.98 (0.80–1.19)
	Boys	315 (15.4)	1896 (84.6)		
Thailand	Girls	324 (22.8)	1066 (77.2)	0.152	0.87 (0.71–1.06)
	Boys	337 (25.4)	1017 (74.6)		
Tonga	Girls	270 (23.2)	896 (76.8)	0.087	1.28 (0.96–1.71)
	Boys	187 (19.1)	794 (80.9)		

OR = Odds Ratio; CI = Confidence Interval; Boldface value are significant at P < 0.05; - no data available.

In the South Asian region, only the Maldives recorded a significant difference between adolescent boys and girls meeting the recommended daily intake of fruit. Specifically, girls were less likely to consume ≥2 servings of fruit or vegetables daily compared to boys. Girls in Pakistan were also less likely to consume the recommended servings of vegetables compared to boys (Tables 1 and 2).

In the Latin American and the Caribbean region, 29% of countries recorded differences (p < 0.05) between adolescent boys and girls in terms of FV intake, particularly in Costa Rica, Dominica, Jamaica, and Peru. More boys met the recommendations for daily fruit intake than the girls in Costa Rica, Dominica and Jamaica. Adolescent girls in Peru on the other hand, were more likely to meet the ≥2 servings of fruit {OR = 1.26; 95%CI (1.05–1.51); p-value = 0.014} than adolescent boys. In Argentina, girls were more likely to have ≥5 servings of FV daily compared to boys {OR = 1.43; 95%CI (1.02–2.00); p-value = 0.037} (Tables 1 and 2).

Lastly, in the East Asian and Pacific region, girls were less likely to meet the daily intake of vegetables compared to boys in Malaysia and Mongolia. Also, in Malaysia, girls were less likely to have ≥5 servings of FV daily {OR = 0.88; 95%CI (0.79–0.96); p-value = 0.007} compared to boys (Tables 1 and 2).

Regarding the daily WHO recommendations for daily PA, differences were observed between adolescent boys and girls in 50% of countries examined in the sub-Saharan African region. In all five countries (Benin, Mauritania, Senegal, Seychelles, and Tanzania), boys were more likely to meet the recommended daily PA guidelines compared to adolescent girls (Table 3).

**Table 3:** Differences in the daily recommended physical activity behavior among adolescent girls and boys in Low-and Middle-Income Countries from the Global School-based Student Health Survey (2004–2013) (N = 163,123 adolescents from 49 LMICs).

**LMICs**		Physical activity recommendations			
		60 mins daily for 7days	60 mins daily for < 7days	Adjusted F (p-value)	OR (95% CI)
		N (weighted %)			
**Sub-Saharan Africa**					
Benin	Boys	476 (27.4)	1257 (72.6)	**0.027**	**1.29 (1.03–1.62)**
	Girls	221 (22.6)	705 (77.4)		
Botswana	Boys	117 (12.6)	825 (87.4)	0.504	1.12 (0.79–1.59)
	Girls	131 (11.4)	1034 (88.6)		
Ghana	Boys	419 (13.5)	2725 (86.5)	0.326	1.11 (0.90–1.36)
	Girls	350 (12.4)	2494 (87.6)		
Malawi	Boys	—	—	—	—
	Girls	—	—		
Mauritania	Boys	158 (16.6)	779 (83.4)	**0.000**	**2.40 (1.68–3.44)**
	Girls	81 (7.6)	960 (92.4)		
Namibia	Boys	289 (10.4)	2513 (89.6)	0.611	0.96 (0.83–1.12)
	Girls	298 (10.8)	2917 (89.2)		
Senegal	Boys	245 (14.3)	1434 (85.7)	**0.000**	**2.47 (1.78–3.45)**
	Girls	90 (6.3)	1297 (93.7)		
Seychelles	Boys	152 (24.3)	481 (75.7)	**0.000**	**2.06 (1.95–2.19)**
	Girls	99 (13.5)	619 (86.5)		
Sudan	Boys	84 (8.8)	748 (91.2)	0.625	1.15 (0.65–2.04)
	Girls	104 (7.7)	1208 (92.3)		
Swaziland	Boys	—	—	—	—
	Girls	—	—		
Tanzania	Boys	290 (27.6)	719 (72.4)	**0.006**	**1.56 (1.16–2.11)**
	Girls	221 (19.6)	878 (80.4)		
Zambia	Boys	78 (8.7)	848 (91.3)	0.150	0.78 (0.56–1.10)
	Girls	98 (10.9)	884 (89.1)		
**Middle East and North Africa**					
Algeria	Boys	504 (23.9)	1622 (76.1)	**0.000**	**3.76 (2.87–4.93)**
	Girls	165 (7.7)	2139 (92.3)		
Djibouti	Boys	183 (18.5)	828 (81.5)	**0.001**	**1.82 (1.32–2.52)**
	Girls	85 (11.1)	674 (88.9)		
Egypt	Girls	242 (19.7)	918 (80.3)	**0.001**	**2.61 (1.51–4.49)**
	Boys	122 (8.6)	1207 (91.4)		
Jordan	Boys	211 (17.5)	997 (82.5)	**0.001**	**1.66 (1.28–2.14)**
	Girls	106 (11.3)	836 (88.7)		
Lebanon	Boys	338 (30.5)	692 (69.5)	**0.000**	**2.21 (1.66–2.94)**
	Girls	202 (16.6)	969 (83.4)		
Libya	Boys	192 (21.5)	727 (78.5)	**0.000**	**2.10 (1.58–2.79)**
	Girls	142 (11.6)	1090 (88.4)		
Morocco	Boys	229 (15.7)	1247 (84.3)	**0.003**	**1.56 (1.20–2.02)**
	Girls	142 (10.7)	1204 (89.3)		
Syria	Boys	174 (14.2)	1053 (85.8)	**0.001**	**1.96 (1.37–2.81)**
	Girls	148 (7.8)	1682 (92.2)		
Tunisia	Boys	352 (26.0)	1003 (74.0)	**0.000**	**2.58 (2.04–3.27)**
	Girls	172 (12.0)	1274 (88.0)		
Yemen	Boys	112 (17.2)	557 (82.8)	0.072	1.62 (0.95–2.78)
	Girls	47 (11.4)	373 (88.6)		
**South Asia**					
India	Boys	1279 (30.3))	3056 (69.7)	0.381	1.10 (0.89–1.35)
	Girls	933 (28.4)	2499 (71.6)		
Maldives	Boys	313 (23.5)	1059 (76.5)	**0.005**	**1.46 (1.12–1.89)**
	Girls	298 (17.4)	1365 (82.6)		
Pakistan	Boys	486 (12.8)	3374 (87.2)	0.564	1.40 (0.42–4.64)
	Girls	116 (9.5)	1148 (90.5)		
Sri Lanka	Boys	194 (17.2)	928 (82.8)	**0.000**	**1.60 (1.28–2.00)**
	Girls	171 (11.5)	1279 (88.5)		
**Latin America and the Caribbean**					
Argentina	Boys	158 (17.3)	773 (82.7)	**0.000**	**2.48 (1.64–3.76)**
	Girls	76 (7.8)	898 (92.2)		
Belize	Boys	236 (24.8)	713 (75.2)	**0.000**	**1.53 (1.26–1.86)**
	Girls	186 (17.7)	883 (82.3)		
Bolivia	Boys	289 (16.6)	1482 (83.4)	**0.000**	**1.60 (1.25–2.07)**
	Girls	180 (11.0)	1537 (89.0)		
Costa Rica	Boys	317 (24.6)	961 (75.4)	**0.000**	**2.38 (1.90–2.98)**
	Girls	162 (12.1)	1212 (87.9)		
Dominica	Boys	113 (16.7)	546 (83.3)	0.545	1.11 (0.79–1.56)
	Girls	132 (15.4)	737 (84.6)		
Grenada	Boys	114 (17.0)	535 (83.0)	0.063	1.35 (0.98–1.84)
	Girls	101 (13.2)	705 (86.8)		
Guatemala	Boys	479 (18.8)	1984 (81.2)	**0.000**	**1.71 (1.31–2.23)**
	Girls	360 (11.9)	2560 (88.1)		
Guyana	Boys	183 (17.9)	827 (82.1)	0.091	1.42 (0.94–2.16)
	Girls	178 (13.3)	1122 (86.7)		
Honduras	Boys	157 (19.3)	664 (80.7)	**0.002**	**1.67 (1.25–2.23)**
	Girls	110 (12.5)	779 (87.5)		
Jamaica	Boys	—	—	—	—
	Girls	—	—		
Peru	Boys	231 (16.4)	1165 (83.6)	0.085	1.20 (0.97–1.47)
	Girls	208 (14.1)	1245 (85.9)		
St. Lucia	Boys	88 (16.4)	441 (83.6)	0.341	1.16 (0.85–1.57)
	Girls	104 (14.5)	617 (85.5)		
St. Vincent and the Grenadines	Boys	95 (15.6)	488 (84.4)	**0.028**	**1.48 (1.05–2.10)**
	Girls	79 (11.1)	593 (88.9)		
Suriname	Boys	187 (22.2)	654 (77.8)	**0.004**	**1.49 (1.05–2.11)**
	Girls	130 (16.0)	679 (84.0)		
**Europe and Central Asia**					
The Former Yugoslav Republic of Macedonia	Boys	176 (18.3)	801 (81.7)	**0.001**	**1.52 (1.21–1.89)**
	Girls	124 (12.9)	899 (87.1)		
**East Asia and Pacific**					
Fiji	Boys	154 (23.7)	540 (76.3)	**0.014**	**1.52 (1.10–2.11)**
	Girls	147 (16.9)	794 (83.1)		
Indonesia	Boys	227 (16.1)	1246 (83.9)	0.706	0.94 (0.69–1.29)
	Girls	263 (16.8)	1352 (83.2)		
Malaysia	Boys	2619 (19.8)	10027 (80.2)	**0.000**	**2.67 (2.41–2.95)**
	Girls	1147 (8.5)	11512 (91.5)		
Mongolia	Boys	754 (30.3)	1737 (69.7)	**0.000**	**1.69 (1.44–1.99)**
	Girls	584 (20.4)	2246 (79.6)		
Myanmar	Boys	251 (19.1)	1142 (80.9)	**0.013**	**1.42 (1.08–1.85)**
	Girls	193 (14.3)	1215 (85.7)		
Philippines	Boys	227 (10.3)	1982 (89.7)	0.050	1.24 (1.00–1.55)
	Girls	235 (8.4)	2682 (91.6)		
Thailand	Boys	290 (21.6)	1066 (78.4)	**0.000**	**2.66 (2.04–3.48)**
	Girls	136 (9.4)	1250 (90.6)		
Tonga	Boys	118 (11.7)	879 (88.3)	**0.018**	**0.70 (0.52–0.94)**
	Girls	181 (15.9)	994 (84.1)		

OR = Odds Ratio; CI = Confidence Interval; Boldface value are significant at P < 0.05; - no data available.

In the Middle East and North African region, almost all the countries recorded a significant difference between boys and girls except for Yemen. In all the countries that showed a statistically significant difference, adolescent boys were more active and were more likely to meet the recommended daily PA guidelines than girls. Also, in the South Asian region, adolescent boys in the Maldives and Sri Lanka were more active than the girls (Table 3).

In the Latin American and the Caribbean region, adolescent boys were more likely to have 60 minutes of daily PA compared to girls, especially in Argentina, Belize, Bolivia, Costa Rica, Guatemala, Honduras, St. Vincent and the Grenadines, and Suriname. In the Former Yugoslav Republic of Macedonia, boys were more active than girls as the results showed boys as being more likely to have 60 minutes of daily PA {OR = 1.52; 95%CI (1.21–1.89); p-value = 0.001} compared to girls (Table 3).

Six of the eight countries (75%) in the East Asian and Pacific region recorded a significant difference between adolescent boys and girls in meeting the daily recommended PA. Five of the six countries (83%) showed that adolescent boys were more likely to have 60 minutes of daily PA for seven days compared to adolescent girls, especially in Fiji, Malaysia, Mongolia, Myanmar, and Thailand. However, in Tonga, adolescent boys were less active, rather the girls were more likely to have 60 minutes of daily PA for seven days {OR = 0.70; 95%CI (0.52–0.94); p-value = 0.018} compared to the boys (Table 3).

## Discussion

This study found that adolescent boys and girls living in LMICs commonly do not meet WHO recommendations for FV intake and physical activity. In all countries, less than 50% of adolescents consumed five servings of FV daily. Morocco had the highest percentage of adolescents meeting the WHO recommendations for the daily intake of FV. According to the FAO^20^, the Moroccan diet is Mediterranean and it is based on a high consumption of FV. Similar findings with respect to the low daily intake of FV among adolescents, have been observed among adolescents in Ghana where 48% rarely ate fruits^7^ and in China where less than 50% ate fruit daily^8^. The variation could be due to differences in dietary cultural preferences among countries or regions as well as the availability of FV in the countries, income and urbanization rate^6^.

The results showed a clear, although variable trend in the FV intake between boys and girls, in which girls were more likely to reach the WHO recommendations. In the case of fruit, 58% of the countries where sex differences were noted showed that girls were more likely to consume ≥2 servings of fruit daily than adolescent boys. On the contrary, for vegetables, 67% of the countries that recorded a sex difference showed that boys were more likely to consume ≥3 servings of vegetables daily than girls. Rasmussen *et al*.^10^, reviewed FV intake in several countries and found that most studies (27 out of 49) showed gender differences in which girls had a higher intake of fruits and/or vegetables. Studies have attributed this gender difference to a preference for FV. It has also been reported that boys do not eat FV because they do not like them as much as girls do^21,22^. In addition to preference, Bere *et al*.^21^ found that the gender differences could also be attributed to the perceived accessibility of FV at home. They found that girls have a greater knowledge, and stronger intentions and self-efficacy compared to boys^21^. Lastly, according to Cooke *et al*.^22^, girls liked fruits and vegetables more than boys. The boys preferred energy-dense foods such as fatty and sugary foods to adapt to their higher energy requirement more so than girls.

According to the WHO^2^, the prevalence of physical inactivity is increasing among LMICs and it is already one of the leading causes of death. Among LMICs, India showed the highest proportion of adolescents who engaged in PA 60 minutes daily. The results from this study are consistent with those from Hallal *et al*.^13^, who found that 80.3% of adolescents between 13–15 years from 105 countries did not meet the recommended daily PA.

The results showed that physical inactivity was more common among adolescent girls in LMICs. In almost all the countries (30 out of 31), boys were more likely to meet the WHO recommendations for PA daily compared to girls. This result is consistent with studies showing that PA declines during adolescence, especially among girls^11,12^. A notable statistical difference between adolescent boys and girls with respect to PA was seen predominantly in the Middle East and North African region. These differences may be attributable to cultural and gender norms. Evidence from the Middle East and North Africa suggest that a barrier for women to engage in PA among women is the lack of facilities separated by gender, where women feel more comfortable being physically active^23,24^.

Despite its design and results, the study had a few limitations. The study included the use of the GSHS data, which surveys adolescents in schools. Thus the results might not necessarily reflect the FV consumption pattern and PA behavior among all adolescents in the respective countries. Specifically, information from adolescents who are unable to attend school or have dropped out would not be captured. Second, in some countries, FV are seasonal staples. Hence their consumption might be high or low in a country based on the season in which the data were collected. As stated earlier, PA patterns may also be affected by cultural perspectives resulting in the differences observed between countries and regions.

Third, adolescents had to recall and self-report their FV consumption and PA behavior during the past 30 days and week, respectively. This approach may result in either overestimating or underestimating intake behaviors, leading to information bias. Finally, we cannot be certain that the 5 servings reported by the adolescents were equivalent to 400 grams since we could not quantify the servings from the GSHS data.

## Conclusion

The WHO recommendations for daily consumption of FV and PA were not consistently met in all countries. All the countries had less than 50% of their adolescents consuming ≥5 servings of FV daily. In general, the study found adolescent boys to be more active than girls, and this difference was more notable in the Middle East and North African region.
